# Tandem repeat distribution of gene transcripts in three plant families

**DOI:** 10.1590/S1415-47572009005000091

**Published:** 2009-12-01

**Authors:** Luciano Carlos da Maia, Velci Queiróz de Souza, Mauricio Marini Kopp, Fernando Irajá Félix de Carvalho, Antonio Costa de Oliveira

**Affiliations:** 1 Centro de Genômica e Fitomelhoramento, Faculdade de Agronomia Eliseu Maciel, Universidade Federal de Pelotas, Pelotas, RSBrazil; 2 Departamento de Agronomia, Universidade Federal de Santa Maria, Frederico Westphalen, RSBrazil; 3 Embrapa Gado de Leite, Juiz de Fora, MGBrazil

**Keywords:** SSR, EST, comparative genomics, molecular markers

## Abstract

Tandem repeats (microsatellites or SSRs) are molecular markers with great potential for plant genetic studies. Modern strategies include the transfer of these markers among widely studied and orphan species. *In silico* analyses allow for studying distribution patterns of microsatellites and predicting which motifs would be more amenable to interspecies transfer. Transcribed sequences (Unigene) from ten species of three plant families were surveyed for the occurrence of micro and minisatellites. Transcripts from different species displayed different rates of tandem repeat occurrence, ranging from 1.47% to 11.28%. Both similar and different patterns were found within and among plant families. The results also indicate a lack of association between genome size and tandem repeat fractions in expressed regions. The conservation of motifs among species and its implication on genome evolution and dynamics are discussed.

## Introduction

Microsatellites or SSRs (*Simple sequence repeats*) are DNA sequences formed by the tandem arrangement of nucleotides through the combination of one to six base pairs, being widely distributed in prokaryote and eukaryote genomes (Morgante and Olivieri, 1993; Tóth *et al.*, 2000). Microsatellite regions tend to form loops or hairpin structures, leading to the slippage of DNA polymerase during replication, thereby provoking the insertion or deletion of nucleotides (Iyer *et al.*, 2000). The expansion and/or contraction of microsatellites may lead to a gain or loss of gene function (Li *et al.*, 2002, 2004a). Initially, it was suggested that the occurrence and distribution of microsatellites could be the result of random processes. However, new evidence indicates that the genomic distribution of these repeats had its origin in non-random processes (Bell, 1996; Li *et al.*, 2004b). Microsatellites have been reported to correspond to 0.85% of Arabidopsis (*Arabidopsis thaliana*), 0.37% of maize (*Zea mays* subsp*. mays*), 3.21% of fugu fish (*Fugu rubripes*), 0.21% of the nematode *Caenorhabditis elegans* and 0.30% of yeast (*Saccharomyces cerevisae*) genomes (Morgante *et al.*, 2002). Moreover, they constitute 3.00% of the human genome (Subramanian *et al.*, 2003).

For microsatellites located in genic regions, 5'UTRs are hotspots for the presence of this type of repeats. It is known that the contraction and/or expansion of repeats found in 5'UTR regions alter the transcription and/or translation of these genes (Li *et al.*, 2004b; Zhang *et al.*, 2006a). Mutations in microsatellite loci found in 3'UTR regions are associated with gene silencing, transcript-cytosol exporting and splicing mechanism changes as well as the expression levels of flanking genes (Davis *et al.*, 1997; Thornton *et al.*, 1997; Philips *et al.*, 1998; Conne *et al.*, 2000). For coding sequences (CDS), the impact of mutations has been described as functional changes, loss of function and protein truncation (Li *et al.*, 2004b). Although much has been reported on microsatellites frequencies in transcribed regions in plants (Temnykh *et al.*, 2001; McCouch *et al.*, 2002; Morgante *et al.*, 2002; Thiel *et al.*, 2003, Nicot *et al.*, 2004; Kashi and King, 2006; Lawon and Zhang, 2006; Varshney *et al.*, 2006; Zhang *et al.*, 2006b), additional comparative or descriptive analysis can offer novel perspectives on their use as molecular markers. The genomic abundance of microsatellites, and their ability to associate with many phenotypes, make this class of molecular markers a powerful tool for diverse application in plant genetics. The identification of microsatellite markers derived from EST and/or cDNAs, and described as functional markers, represents an even more useful possibility for these markers when compared to those based on assessing anonymous regions (Varshney *et al.*, 2005, 2006).

In order to provide information regarding the patterns of microsatellite occurrence and distribution on transcribed genome regions, non-redundant full-length cDNAs (*fl*-cDNAs) and/or ESTs belonging to ten plant species from three different families (Brassicaceae, Solanaceae and Poaceae) were used.

## Material and Methods

###  Obtaining the expressed sequence

Files containing expressed sequences were obtained for the following families/species: Brassicaceae (*Arabidopsis thaliana* and *Brassica napus),* Solanaceae (*Solanum lycopersicum* and *Solanum tuberosum*) and Poaceae (*Oryza sativa, Sorghum bicolor, Triticum aestivum, Zea mays, Saccharum officinarum* and *Hordeum vulgare)*, all deposited in the NCBI*-*Unigene data-base. Non-redundant yet representative sequences for all known genes in each species were selected. The sequences used in the present study were downloaded from the Unigene database in June, 2008.

###  Distribution of sequences in different transcribed regions

By using computer scripts developed in Perl language and based on the existing annotation for each of the cDNAs and/or ESTs sequences, the sequences were categorized as CDS, upstream and downstream regions, partitioned into fasta files and denominated CDS, 5' UTR and 3' UTR for each species. Since the annotation of introns was not part of the database, the repeats present in intronic regions were not considered in this study.

###  Location of tandem repeats

*SSRLocator* software was used (Maia *et al.*, 2008) for the location of tandem repeats. Software options were adjusted to locate monomers, dimers, trimers, pentamers and hexamers containing a minimum of 10, 7, 5, 4 and 4 repeats, respectively. For mini-satellites, heptamer, octamer, nonamer and decamers containing a minimum of 3, 3, 3 and 2 repeats, respectively, were selected.

## Results and Discussion

###  Distribution of sequences in UTRs and CDSs

The sequences, separated into coding (CDS) and untranslated (5'UTR and 3'UTR) regions, and distributed by number of sequences, amount (Mb) and average size (bp) for all the ten species, are shown in Table 1. On an average and in all of these, there were sequence fragments between 560 and 893 bp long, except for the *A. thaliana* and *O. sativa* databases*,* where they were longer, reaching averages of 1,447 and 1,490 bp, respectively. The number of sequences deposited in Unigene was the largest for both of the Poaceae species *Z. mays* and *O. sativa*, with 57,447 and 40,259, respectively. It is worthy of note that not all sequences deposited in this database contain 5'UTR and 3'UTR regions, for in some both types are found, whereas in others only one is (*i.e.*, 5' or 3'UTR). The overall average sizes were found to be 130 bp for 5'UTR, 873 bp for CDS and 270 bp for 3'UTR regions. The total nucleotides allocated to each were, on an average, 0.9% for 5'UTR, 97.5% for CDS and 1.6% for 3'UTR. The only species with contrasting values was Arabidopsis, where 6.8%, 82.6% and 10.7% of total nucleotides were allocated to 5'UTR, CDS and 3'UTR regions, respectively.

###  Percentage of expressed sequences with tandem repeats

**Figure 1:**
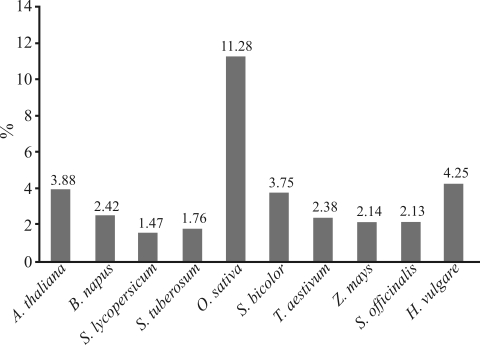
Percentage of expressed sequences containing tandem repeat loci.

On an average, 3.55% of analyzed sequences contain one or more loci with tandem repeats. The respective percentages for each species are shown in Figure 1. The highest were for rice (11.28%), and the lowest for the Solanaceae species *S. lycopersicum* and *S. tuberosum*, *i.e.*, 1,47% and 1,76%, respectively. The percentage found for *Arabidopsis* (3.88%) is in agreement with other reports of between 3% and 5% (Cardle *et al.*, 2000; Kumpatla and Mukhopadhyay, 2005). For *B. napus*, *S. lycopersicon* and *S. tuberosum* 2.42%, 1.47% and 1.76% of these sequences were found, respectively. However, different values (6.9%, 4.7% and 2.65%, respectively) have been reported (Kumpatla and Mukhopadhyay, 2005). For the Poaceae, a comparison of present results with former reports for *H. vulgare* (4.25% *vs.* 8.11%)*, Z. mays* (2.14% *vs.* 1.5%), *O. sativa* (11.28% *vs.* 4.7%), *S. officinarum* (2.13% *vs.* 2.9%) and *T. aestivum* (2.38% *vs.* 7.5%) show a different range of values (Cordeiro *et al.*, 2001; Kantety *et al.*, 2002; Thiel *et al.*, 2003; Nicot *et al.*, 2004; Asp *et al.*., 2007). Nevertheless, all differences are within the 2-3 fold range.

The variations encountered in different reports are related to the strategy employed by the authors (software, repeat number and type defined for the search). However, by common agreement, microsatellite stretches with minimum sizes of 20 bp are present in approximately 2%-5% of cereal EST sequences (Varshney *et al.*, 2005).

###  Frequency of tandem repeats in UTR and CDS regions

Results for total occurrence (total loci), percentage per region (the amount of loci per region divided by their total number) and frequencies (amount of loci per megabase) are shown separately for each species and by genic region (5'UTR, CDS and 3'UTR) in Table 2. In the 5'UTR and 3'UTR regions, 4.92% (529 loci) and 2.21% (237 loci), respectively, of all repeats were found in all the surveyed species (10,731 loci), with an average frequency of 1.3 and 0.7 loci/Mb, respectively. In coding regions (CDS), a higher occurrence of micro and minisatellites was detected, this reaching 92.86% of the total loci found (9,965 occurrences) with an average frequency of 35.1 loci/Mb. The higher percentage of repeats occurred in CDS regions as a consequence of the trimers present in this region. However, for *Arabidopsis*, high percentages of dimer (17.9%), trimer (19.3%) and total (44.5%) microsatellites were found in UTR regions, thus contrasting with the other species (Table 3). For the Rosaceae, between 44.3% and 53.2% of the microsatellites were found in UTR regions (Jung *et al.*, 2005). For *Arabidopsis*, 81% and 26%, respectively, of dimers and trimers were found in UTR regions (Yu *et al.*, 2004).

In the present study, a very high percentage of microsatellites in 5'UTRs were detected in *Arabidopsis,* with a frequency of 9.1 loci/Mb. These repeats represented 34% of all the 1,162 found in the 29,918 sequences analyzed in this species. The second and third highest frequencies of repeats in these regions were encountered in the species *O. sativa* and *H. vulgare*, with an average 1.3 and 1.0 loci/Mb, respectively (Table 2).

Many studies indicate the UTR regions as being more abundant in microsatellites than CDS regions (Morgante *et al.*, 2002). In the present work, 92.86% of microsatellite loci in CDS regions are due to a deficiency in annotation when separating translated from non-translated fractions in the Unigene transcript database.

As observed for 5'UTRs, contrasting values were also found in 3'UTR regions. Much higher values were encountered in *Arabidopsis* (an average of 3.6 loci/Mb) when compared to those below 0.6 loci/Mb in the remaining species (Table 2).

On considering the overall occurrence of 5'UTRs, 3'UTRs and CDSs in all species, the average frequency observed is 37 loci/Mb. Values normally range from 18 loci/Mb in tomato to 76 in rice. Average frequency values per family are 29.0 loci/Mb in the Brassicaceae, 19.9 in the Solanaceae and 45.4 in the Poaceae (Table 2).

Several reports have indicated values higher than those found in this study, *i.e.*, 112-133 loci/Mb in barley, 133 loci/Mb in maize, 94-161 loci/Mb in wheat, 158-169 loci/Mb in sorghum, 161 loci/Mb in rye, 256-277 loci/Mb in rice and 133 loci/Mb in *Arabidopsis* (Varshney *et al.*, 2002; Thiel *et al.*, 2003; Parida *et al.*, 2006). In Citrus species, values as high as 507 loci/Mb have been described in EST sequences (Palmieri *et al.*, 2007). Values as high as 125 loci/Mb were also noted in *Brassica rapa* (Hong *et al.*, 2007). Frequency values closer to our study have been reported for the CDS regions in *Rosa chinensis* (Rose), *Prunus dulcis* (Almond), *Prunus persica* (Peach) and *Arabidopsis*, with values ranging from 39 to 78 loci/Mb (Jung *et al.*, 2005)*.*

###  Percentage occurrence of different microsatellite types in the UTR and CDS regions

The detailed percentage values for each repeat type in the diverse sections of a genic region are listed for each species in Table 3. The average occurrence of dimer microsatellites in all the species was 21.9%, the majority of these loci being present in the CDS regions. The average percentage of dimer occurrence for each family was 31.5% in Brassicaceae, 21.7% in Solanaceae and 18.8% in Poaceae species. The percentage values for dimer microsatellites in CDS regions ranged from 4.0% in *Arabidopsis* to 40.8% in *B. napus.* An interesting feature which seems to be specific for the *Arabidopsis* genome is the high occurrence of dimer microsatellites in the 5' and 3' UTR regions (13,6% and 4,3%, respectively). In the Poaceae, dimer microsatellites ranged from 15.4% in barley to 27.3% in wheat (Table 3). Other studies indicated that the highest dimer occurrence rates are generally associated with 5'UTR regions (Morgante *et al.*, 2002; Lawson and Zhang, 2006; Hong *et al.*, 2007), but one should bear in mind that this prevalence in CDS regions may be a consequence of deficient database annotation. Trimer microsatellites were found in 40.2% of the sequences, with a high predominance in CDS regions. The species with higher trimer values were *Arabidopsis*, rice and tomato, with 58.0%, 54.7% and 41.4% of occurrence, respectively. The average percentage of trimers within each family was 47.0% in the Brassicaceae, 37.8% in the Solanaceae and 38.7% in the Poaceae*.* Among Poaceae species, the highest percentage of trimer occurrence was found in rice (54.7%) and the lowest in maize (34.6%). In Brassicaceae, trimers were found more frequently in *Arabidopsis* (58.0%) and less so in *B. napus* (36.1%) (Table 3).

On an average, tetramers represented 8.2% of the microsatellites, with average frequencies of 3.4%, 4.4% and 11.0% in Brassicaceae, Solanaceae and Poaceae, respectively. Among the Brassicaceae, a less than one-fold difference in frequencies was observed between *Arabidopsis* (2.9%) and *B. napus* (4.4%). In Poaceae, a 2.7-fold difference was found between rice (6.1%) and barley (16.5%).

On an average, pentamers represented 10.36% of the microsatellites, with average frequencies of 4.5%, 6.6% and 13.6% in the Brassicaceae, Solanaceae and Poaceae, respectively (Table 3). Less than one-fold differences were found between Brassicaceae and Solanaceae species. Nevertheless, in the Poaceae a 1.7-fold difference was found between rice (9.7%) and maize (16.5%).

On an average, hexamers represented 13.8% of the microsatellites, with average frequencies of 8.1%, 19.1% and 13% in the Brassicaceae, Solanaceae and Poaceae, respectively. In the Poaceae, a 2.4-fold difference was found between wheat (7.7%) and sorghum (18.3%).

Mini-satellite frequencies were also assessed from the available data (Table 3). On an average, heptamers represented 4.5% of the total occurrence (mini-satellite plus microsatellite). These types of repeats were more common in the Solanaceae family (9.6%). In both the Brassicaceae and Poaceae, the average frequencies of heptamers were 3.3% and 3.2%, respectively. Octamers were more frequent in the Brassicaceae (0.8%), when compared to the Solanaceae (0.3%) and Poaceae (0.1%). Nonamers were also more frequent in the Brassicaceae (0.9%), when compared to the Solanaceae (0.6%) and Poaceae (0.5%). Decamers were comparatively less frequent than other mini-satellites, reaching frequencies of 0.2%, 0.1% and zero in the Brassicaceae, Poaceae and Solanaceae, respectively (Table 3).

There are several studies proclaiming EST sequences containing microsatellites. For the Poaceae (rice, maize, sorghum, barley and wheat), frequencies ranging from 16.6 to 40% for dimers, 41 to 78% for trimers, 2.6 to 14% for tetramers, 0.4 to 18.9% for pentamers and below 1% for hexamers (Varshney *et al.*, 2002; Thiel *et al.*, 2003; La Rota *et al.*, 2005; Parida *et al.*, 2006) have been reported. In the case of *Arabidopsis*, frequencies of dimers (36.5%), trimers (62.1%), tetramers (1.1%), pentamers (0.15%) and hexamers (0.13%) have been noted (Parida *et al.*, 2006).

###  Most frequent motifs

####  Dimers and trimers 

Motif frequencies per species and average frequency per family are listed in Tables 4  and 5. For dimers, differences were observed within and between families. As regards the Brassicaceae, AG/CT and GA/TC dimer motifs were the most frequent, reaching 9.69% and 8.89% of observations within the family. A 6.9-fold difference was the case for AG/CT between *Arabidopsis* (2.46%) and *B. napus* (16.93%). Moreover, as to the GA/TC motif, an almost 10-fold difference was found between *Arabidopsis* (1.64%) and *B. napus* (16.14%). Other reports have shown that AG/GA motifs were the most frequent in *Arabidopsis* (Cardle *et al.*, 2000; Morgante *et al.*, 2002; Lawson and Zhang, 2006; Parida *et al.*, 2006) and AT/TA in *B. rapa* (Hong *et al.*, 2007). Among the Solanaceae, AT/AT and TA/TA motifs were the most frequent, with frequencies of 8.29% and 5.69%, respectively. In Solanaceae ESTs, frequencies between 20%-25% and 15%-20% were found for AG and AT dimers, respectively (Kumptla and Mukhopadhyay, 2005). In the Poaceae, the most frequent motifs were AG/CT and GA/TC, with average percentages of 6.72% and 5.61%, respectively. In still other studies, frequencies ranging from 38%-50% were the rule for the AG motif in maize, barley, rice, sorghum and wheat (Kantety *et al.*, 2002; Morgante *et al.*, 2002; Varshney *et al.*, 2002; Thiel *et al.*, 2003; Yu *et al.*, 2004; La Rota *et al.*, 2005) and frequencies of 50% for the AC motif in barley (Varshney *et al.*, 2002). GA has also been shown to be the most abundant motif in grasses (Temnykh *et al.*, 2001; Kantety *et al.*, 2002; Nicot *et al.*, 2004; Parida *et al.*, 2006). In all the species that were analyzed in the present study, the lowest frequencies were found for those motifs formed by guanine and cytosine (CG/GC), which were even absent in Brassicaceae and Solanaceae species.

As was the case for dimers, in trimer frequencies motif patterns are different within as well as between families (Table 4). Among the Brassicaceae, GAA/TTC and AAG/CTT motifs were the most abundant, reaching frequencies of 8.36% and 6.73%, respectively. Contrasting values were verified for GAA/TTC between *Arabidopsis* (12.13%) and *B. napus* (4.59%), also the case for AAG/CTT between *Arabidopsis* (9.51%) and *B. napus* (3.96%). Some reports have claimed that AAG is the most frequent for *Arabidopsis* and *B. rapa* (Morgante *et al.*, 2002; Hong *et al.*, 2007). In the Solanaceae*,* GAA/TCC and AGA/TCT were the most frequent, with values of 4.75% and 4.60%, respectively. For both, frequency values were higher in *S. tuberosum*. Similar results were obtained in *Arabidopsis, B. napus, B.rapa, S. Lycopersicum* and *S. tuberosum* (Kumptla and Mukhopadhyay, 2005), as well as in *Citrus* (Jiang *et al.*, 2006) where AAG/AGA/GAA motifs were the most frequent. In the Poaceae, the trimers CCG/CGG, CGC/GCG and GCC/GGC were the most frequent, corresponding to 5.89%, 5.85% and 5.06%, respectively, a total of 16.80% of all the microsatellites found. Within the family, different motifs were the most common, *i.e.*, for *O. sativa*, *S. bicolor* and *H. vulgare*, CCG/CGG were predominant, for *T. aestivum* and *S. officinarum* GCC/GGC and for *Z. mays* CGC/GCG. Other studies have shown a predominance of CCG in the grass species *Z. mays, H. vulgare*, *O. sativa, S. bicolor, T. aestivum, S. cereale* and *S. officinarum* (Cordeiro *et al.*, 2001; Kantety *et al.*, 2002; Morgante *et al.*, 2002; Varshney *et al.*, 2002; Thiel *et al.*, 2003; Nicot *et al.*, 2004; Yu *et al.*, 2004; La Rota *et al.*, 2005; Peng and Lapitan*,* 2005). These motifs (CCG/CGG, CGC/GCG and GCC/GGC) seem to be less common in other families, where instead of values of around 16.8% (found for grasses), frequency was 0.56% in Brassicaceae and 0.36% in the Solanaceae.

####  Tetramers, pentamers and hexamers 

For the loci formed by motifs longer than three nucleotides, only the ten highest average percentages for each family are shown (Tables 4  and 5).

In Brassicaceae, tetramer motifs occurring at higher frequencies were AAGA/TCTT, AAAC/GTTT or GAAA/TTTC adding to 1.04% of all motifs found. Other reports indicate that motifs AAAG/AAAT were predominant in *Arabidopsis* and AAAT in *B. rapa* (Cardle *et al.*, 2000; Hong *et al.*, 2007). For 5'UTR/CDS and 3'UTR *Arabidopsis* regions, the predominant motifs reported were AAAG/CTTT and AAAC/GTTT, respectively (Morgante *et al.*, 2002; Zhang *et al.*, 2004). For Solanaceae species, 1.96% of all motifs found were either TAAA/TTTA or TTAA/TTAA or AAGA/TCTT. These results agree with EST data from 20 dicot species (Kumptla and Mukhopadhyay, 2005). Among the grasses, 0.85% of all motifs were either CCTC/GAGG or AGGA/TCCT or CATC/GATG. Differences in predominant tetramer rates were found among the species (Table 4). Other reports have shown ACGT as the most abundant in barley (Varshney *et al.*, 2002; Thiel *et al.*, 2003), AAAG/CTTT and AAGG/CCTT in perennial ryegrass (Asp *et al.* 2007) and AAAG as the most frequent motif in rice BACs (McCouch *et al.*, 2002).

For pentamers, 0.80% (GAAAA/TTTTC, AAAAT/ ATTTT and AAAAC/GTTTT), 1.37% (AAAAT/ATTTT, AAAAG/CTTTT and AGAAG/CTTCT) and 0.83% (CTCTC/GAGAG, GAGGA/TCCTC and CTTCC/ GGAAG) were predominant in the Brassicaceae, Solanaceae and Poaceae, respectively. The major difference among plant families is the predominance of A/T in the Brassicaceae and Solanaceae. Also, reports on CDS regions in *Arabidopsis, S. cerevisae* and *C.elegans*, indicated the predominance of ACCCG and AAAAG (Toth *et al.* 2000). For eukaryotes in general, AAAAT, AAAAC and AAAAG are revealed as the most predominant (Li *et al.*, 2004a). On the other hand, 5'UTR and 3'UTR regions of *Arabidopsis* were shown to be rich in AAGAG and AAAAC, respectively (Zhang *et al.*, 2004). AAAAT (Hong *et al.*, 2007) and AAAAT /AAAAG (Jiang *et al.*, 2006) were described as being frequently found in the Rosaceae and Citrus, respectively. In transcripts from the TIGR database, the AGAGG motif was predominant in rice, AGGGG in barley and ACGAT in wheat (La Rota *et al.*, 2005). Very little information was encountered on the preferential occurrence of pentamers in grasses, whereas that on eukaryotes (Toth *et al.*, 2000; Li *et al.*, 2004a), *Citrus* (Palmieri *et al.*, 2007; Jiang et *al.*, 2006), *Arabidopsis* (Zhang et *al.*, 2004) and Rosaceae (Hong *et al.*, 2007) offered variable results.

Hexamer patterns occurred among and within the three analyzed plant families (Table 5). To date, the predominance of AAGGAG hexamers in *Arabidopsis*, has been confirmed by only one other study (Toth *et al.*, 2000). Other reports indicated the most encountered hexamers to be AAGATG, AAAGAG and AAAAAT in *Arabidopsis* (Zhang *et al.*, 2004), AAAAAG in *Citrus* (Jiang *et al.*, 2006), AACACG in *S. cerevisae,* ACCAGG in *C. elegans*, AAGGCC in mammals and CCCCGG in primates (Toth *et al.*, 2000). The ten major occurrences for heptamers, octamers, nonamers and decamers are presented in Table 5. Occurrences are widely variable within and among families, making it difficult to establish either a pattern or discussion based on similarities.

Genome dynamics is very complex regarding microsatellite motifs in plants. The higher conservation of dimer motifs (AG/TC and GA/TC) seems to overcome evolutionary barriers distances such as those found between monocot and dicot plants. However, in the dicots, this conservation may not hold. Unexpectedly, Poaceae and Brassicaceae were closer when these motifs were analyzed. On the other hand, trimer microsatellites that are known to be predominant in coding regions followed the expected conservation pattern, with similar rates and predominant motifs (GAA/TTC) between the two dicot families. Trimers present at higher frequencies in the grasses tend to be formed by G/C arrangements, in contrast to dicot plants where G/A/T/C combinations are more frequent. The higher frequency of A/T- rich repeats is also found in pentamer motifs in the dicot families. Repeats of higher complexity did not reveal detectable conserved patterns in this study.

## Conclusions

The occurrence of micro and minisatellites in rice sequences (11.28%) is higher than in other species, ranging from 2.5 to 5 times more sequences containing these repetitive DNA loci. The fact that species having larger genomes (*T. aestivum*, *H. vulgare* and *S. officinarum*) do not present a correspondingly higher frequency of repetitive loci implies there is no relationship between genome size and rates of tandem repeat occurrence in functional regions. However, the lower coverage of sequences present in databases for these species could also be a reason for the low rates found in some species. For *Arabidopsis* and rice, the results obtained are closer to reality, since both are considered model species and have been intensely studied.

The distribution of micro- and minisatellites was higher in CDS regions for all the studied species. Also, microsatellites (97%) were more common than mini-satellites (3%). Per family, the predominant dimer motifs were the same for Brassicaceae and Poaceae (AG/CT) and different for the Solanaceae (AT/AT). Trimers were the predominant repeats, ranging between 34.3% and 58.0%, with different rates depending on the family or species. For the Solanaceae, the predominant trimer motifs were not the same for *S.lycopersicum* (ATA/TAT and AAT/TTA) and *S. tuberosum* (GAA/TTC and AGA/TCT). This could be due to selection. Among the grasses, trimers formed by C/G were the most abundant. Nevertheless, specific motifs were variable between species.

Disagreements between earlier reports and the results obtained in the present work, where dimers were also frequent in CDS regions, could be due to the fact that the Unigene database contains predominantly EST clusters. Therefore, there is a tendency for under-representing the UTR regions in the annotated sequences. This is true for all species, except *Arabidopsis.* This could be solved by manually curating the genes, thereby defining the different regions. Achievement, however, would require a community effort.

The obtained results shed light on the patterns of tandem repeat occurrence within and between different plant families, thereby facilitating the use of plant-breeding strategies based on the transfer of markers from model to orphan species.

## Figures and Tables

**Table 1:** Overall distribution (amounts and percentage) of expressed sequences in translated and non-translated regions.

	Expressed sequences				5' UTR				CDS				3' UTR		
	Total seq.^1^	Total mb^1^	Mean pb^1^		Total seq.^2^	% mb^2^	Mean pb^2^		Total seq.^3^	% mb^3^	Mean pb^3^		Total seq.^4^	% mb^4^	Mean pb^4^
*A. thaliana*	29,918	43.3	1,447		16,625	6.8	176		29,918	82.6	1,195		17,591	10.7	262
*B. napus*	26,285	20.3	773		216	0.1	74		26,285	99.7	770		242	0.2	204
*S. lycopersicum*	16,945	14.0	823		614	0.5	103		16,945	98.3	809		710	1.2	245
*S. tuberosum*	19,539	15.6	796		554	0.3	93		19,539	98.6	785		635	1.0	252
*O. sativa*	40,259	60.0	1,490		1,088	0.5	270		40,259	98.7	1,470		1,158	0.8	438
*S. bicolor*	13,547	9.5	699		68	0.1	115		13,547	99.7	697		82	0.2	244
*T. aestivum*	34,505	26.2	758		498	0.2	92		34,505	99.2	753		611	0.6	246
*Z. mays*	57,447	32.2	560		704	0.3	120		57,447	99.1	555		803	0.7	275
*S. officinarum*	15,586	12.7	815		48	0.1	160		15,586	99.8	813		54	0.1	273
*H. vulgare*	21,418	19.1	893		359	0.2	102		21,418	99.2	886		458	0.6	259
Averages	27,545	25.3	905		2,077	0.9	130		27,545	97.5	873		2,234	1.6	269.8

Expressed sequences: Total Seq.^1^ (Total number of cDNA sequences), Total mb^1^ (sum of base pairs of fl-cDNA sequences), Mean pb^1^ (average size of sequences – sum of base pairs divided by number of sequences (Total mb^1^ / Total Seq.^1^)). 5'UTR: Total Seq.^2^ (Total sequences containing 5'UTR regions), % mb^2^ (percentage of Total mb^1^ contained in 5'UTR regions), Mean pb^2^ (average size of 5'UTR sequences- sum of base pairs divided by the number of sequences (Total pb(% mb^2^) / Total Seq.^2^)). CDS: Total Seq.^3^ (Total sequences containing CDS regions), % mb^3^ (percentage of Total mb^1^ contained in CDS regions), Mean pb^3^ [average size of CDS sequences – sum of base pairs divided by number of sequences (Total pb(% mb^3^) / Total Seq.^3^)]. 3'UTR: Total Seq.^4^ (Total of sequences containing 3'UTR regions), % mb^4^ (percentage of Total mb^1^ contained in 3'UTR regions), Mean pb^4^ (average size of 3'UTR sequences – sum of base pairs divided by the number of sequences (Total pb(% mb^4^) / Total Seq.^4^)).

**Table 2:** Overall distribution of tandem repeat occurrences in translated and non-translated transcripts.

	5' UTR				CDS				3' UTR				Total	
	Occurrence	%	ssr/Mb		Occurrence	%	ssr/Mb		Occurrence	%	ssr/Mb		Occurrence	ssr/Mb
*A. thaliana*	395	34.0	9.1		610	52.5	14.1		157	13.5	3.6		1,162	27
*B. napus*	1	0.2	0.0		632	99.5	31.1		2	0.3	0.1		635	31
*S. lycopersicum*	6	2.4	0.4		234	94.0	16.8		9	3.6	0.6		249	18
*S. tuberosum*	4	1.2	0.3		336	97.7	21.6		4	1.2	0.3		344	22
*O. sativa*	78	1.7	1.3		4,433	97.6	73.9		29	0.6	0.5		4,540	76
*S. bicolor*	3	0.6	0.3		505	99.4	53.3		0	0.0	0.0		508	54
*T. aestivum*	11	1.3	0.4		795	97.0	30.4		14	1.7	0.5		820	31
*Z. mays*	12	1.0	0.4		1,205	98.0	37.4		13	1.1	0.4		1,230	38
*S. officinarum*	0	0.0	0.0		332	100.0	26.1		0	0.0	0.0		332	26
*H. vulgare*	19	2.1	1.0		883	96.9	46.2		9	1.0	0.5		911	48
Average	529	4.9	1.3		9,965	92.9	35.1		237	2.2	0.7		10,731	37

**Table 3:** Overall occurrence, in percentage, of microsatellite and minisatellite motifs in different sections of genic regions of ten plant species.

	Dimer					Trimer					Tetramer					Pentamer					Hexamer			
Microssatélites	5'UTR	CDS	3'UTR	Total		5'UTR	CDS	3'UTR	Total		5'UTR	CDS	3'UTR	Total		5'UTR	CDS	3'UTR	Total		5'UTR	CDS	3'UTR	Total
*A. thaliana*	13.6	4.0	4.3	21.9		14.6	38.6	4.7	58.0		1.0	0.9	1.0	2.9		2.1	0.8	1.9	4.7		0.9	5.8	0.4	7.1
*B. napus*	0.2	40.8	0.2	41.2		-	35.9	0.2	36.1		-	4.4	-	4.4		-	4.3	-	4.3		-	9.1	-	9.1
*S. lycopersicum*	0.4	17.7	2.0	20.1		0.4	40.2	0.8	41.4		-	4.4	-	4.4		-	6.0	0.8	6.8		0.8	17.3	-	18.1
*S. tuberosum*	0.3	22.4	0.6	23.3		0.3	34.0	-	34.3		-	4.4	-	4.4		-	6.1	0.3	6.4		-	20.1	-	20.1
*O. sativa*	0.5	14.9	0.3	15.7		0.7	53.9	0.1	54.7		0.0	6.0	0.1	6.1		0.3	9.3	0.1	9.7		0.1	10.3	0.0	10.4
*S. bicolor*	0.2	18.5	-	18.7		0.2	35.2	-	35.4		-	10.2	-	10.2		-	14.6	-	14.6		0.2	18.1	-	18.3
*T. aestevum*	0.5	26.5	0.4	27.3		0.5	34.0	0.5	35.0		0.2	13.3	0.1	13.7		0.1	11.3	0.6	12.1		-	7.6	0.1	7.7
*Z. mays*	0.5	16.0	0.5	17.0		0.2	34.5	-	34.6		0.1	10.7	0.4	11.2		0.1	16.2	0.2	16.4		0.1	17.4	-	17.5
*S. officinarum*	-	18.7	-	18.7		-	36.4	-	36.4		-	8.4	-	8.4		-	14.5	-	14.5		-	16.9	-	16.9
*H. vulgare*	0.5	14.6	0.2	15.4		0.7	35.1	0.3	36.1		0.4	15.7	0.3	16.5		0.2	13.8	0.1	14.2		0.1	12.8	-	13.0
Average	1.7	19.4	0.8	21.9		1.7	37.8	0.7	40.2		0.2	7.8	0.2	8.2		0.3	9.7	0.4	10.4		0.2	13.5	0.1	13.8

	Heptamer					Octamer					Nonamer					Decamer					General			
Minissatélites	5'UTR	CDS	3'UTR	Total		5'UTR	CDS	3'UTR	Total		5'UTR	CDS	3'UTR	Total		5'UTR	CDS	3'UTR	Total		5'UTR	CDS	3'UTR	Total

*A. thaliana*	1.0	0.9	0.8	2.8		0.6	0.3	0.3	1.2		0.1	1.0	-	1.1		0.1	0.2	0.1	0.3		34.0	52.5	13.5	100.0
*B. napus*	-	3.8	-	3.8		-	0.5	-	0.5		-	0.6	-	0.6		-	0.2	-	0.2		0.2	99.5	0.3	100.0
*S. lycopersicum*	0.8	8.4	-	9.2		-	-	-	-		-	-	-	-		-	-	-	-		2.4	94.0	3.6	100.0
*S. tuberosum*	0.6	9.0	0.3	9.9		-	0.6	-	0.6		-	1.2	-	1.2		-	-	-	-		1.2	97.7	1.2	100.0
*O. sativa*	0.1	2.0	0.0	2.1		0.0	0.2	-	0.2		-	0.7	-	0.7		-	0.3	-	0.3		1.7	97.6	0.6	100.0
*S. bicolor*	-	2.4	-	2.4		-	-	-	-		-	0.4	-	0.4		-	-	-	-		0.6	99.4	-	100.0
*T. aestevum*	-	3.5	-	3.5		-	0.2	-	0.2		-	0.4	-	0.4		-	0.1	-	0.1		1.3	97.0	1.7	100.0
*Z. mays*	0.1	3.0	-	3.1		-	-	-	-		-	0.2	-	0.2		-	-	-	-		1.0	98.0	1.1	100.0
*S. officinarum*	-	4.2	-	4.2		-	-	-	-		-	0.6	-	0.6		-	0.3	-	0.3		-	100.0	-	100.0
*H. vulgare*	0.1	3.6	-	3.7		-	0.2	-	0.2		-	1.0	-	1.0		-	-	-	-		2.1	96.9	1.0	100.0
Average	0.3	4.1	0.1	4.5		0.1	0.2	0.0	0.3		0.0	0.6	-	0.6		0.0	0.1	0.0	0.1		4.4	93.3	2.3	100.0

**Table 4:** Distribution of di-, tri- and tetramer motifs, percentage occurrence per species and average occurrence per family.

	Brassicaceae					Solanaceae					Poaceae						
	Ara	Bra	Average			Lyc	Sol	Average			Ory	Sor	Tri	Zea	Sac	Hor	Average
Dimers																	
AG/CT	2.46	16.93	9.69		AT/AT	8.55	8.04	8.29		AG/CT	6.38	5.15	9.06	6.56	6.63	6.57	6.72
GA/TC	1.64	16.14	8.89		TA/TA	5.13	6.25	5.69		GA/TC	5.46	5.35	10.19	5.15	3.92	3.62	5.61
AT/AT	1.80	4.11	2.96		GA/TC	1.71	4.76	3.24		AT/AT	1.31	1.39	1.01	1.83	2.71	1.25	1.58
TA/TA	0.98	2.22	1.60		AG/CT	3.42	2.98	3.20		CA/TG	0.56	2.38	2.89	0.75	1.51	1.36	1.57
GT/AC	0.49	0.79	0.64		GT/AC	0.00	0.60	0.30		GT/AC	0.59	2.38	2.77	0.50	1.20	1.36	1.47
CA/TG	0.16	0.79	0.48		CA/TG	0.00	0.30	0.15		TA/TA	0.92	1.98	1.26	1.58	2.41	0.57	1.45
GC/GC	0.00	0.00	0.00		GC/GC	0.00	0.00	0.00		GC/GC	0.00	0.00	0.00	0.00	0.30	0.23	0.09
CG/CG	0.00	0.00	0.00		CG/CG	0.00	0.00	0.00		CG/CG	0.07	0.00	0.13	0.00	0.00	0.11	0.05

Trimers																	
GAA/TTC	12.13	4.59	8.36		GAA/TTC	3.85	5.65	4.75		CCG/CGG	11.41	5.15	2.52	5.81	4.22	6.23	5.89
AAG/CTT	9.51	3.96	6.73		AGA/TCT	3.85	5.36	4.60		CGC/GCG	10.47	4.75	3.02	5.98	6.02	4.87	5.85
AGA/TCT	8.85	4.59	6.72		ATA/TAT	5.13	3.57	4.35		GCC/GGC	6.11	4.95	3.27	5.81	6.93	3.28	5.06
ATC/GAT	7.54	2.22	4.88		AAT/ATT	4.27	2.98	3.62		CAG/CTG	1.87	2.77	2.64	2.41	3.31	2.60	2.60
TCA/TGA	4.59	2.37	3.48		AAG/CTT	3.42	3.57	3.50		GCA/TGC	1.47	2.77	2.01	2.16	1.81	2.83	2.17
CAA/TTG	4.75	1.90	3.33		TAA/TTA	2.99	1.19	2.09		CTC/GAG	3.77	1.19	1.89	1.49	2.41	2.15	2.15
ATG/CAT	4.43	1.74	3.08		CAA/TTG	2.14	1.19	1.66		AGC/GCT	1.47	2.18	1.26	1.16	2.41	2.27	1.79
AAC/GTT	4.10	1.27	2.68		CTC/GAG	2.14	0.60	1.37		AGG/CCT	2.50	1.19	1.89	1.24	0.30	1.59	1.45
ACA/TGT	3.93	1.11	2.52		CAG/CTG	2.14	0.60	1.37		GGA/TCC	2.57	0.99	1.13	1.74	1.20	0.79	1.41
GGA/TCC	3.44	0.79	2.12		TCA/TGA	0.85	1.79	1.32		AAG/CTT	1.51	0.59	1.64	0.41	0.30	1.59	1.01
AGG/CCT	1.31	2.06	1.68		ACA/TGT	1.71	0.89	1.30		CAA/TTG	0.29	0.40	3.02	0.41	1.20	0.68	1.00
CTC/GAG	1.15	2.22	1.68		CAC/GTG	2.14	0.30	1.22		CCA/TGG	1.38	1.39	0.38	0.75	0.60	1.13	0.94
ACC/GGT	2.13	0.63	1.38		ATC/GAT	1.71	0.60	1.15		CGA/TCG	1.58	0.99	0.38	1.58	0.30	0.34	0.86
CCA/TGG	1.48	1.11	1.29		CCA/TGG	0.85	1.19	1.02		CAC/GTG	0.99	0.79	0.75	0.58	0.90	1.13	0.86
CAC/GTG	1.31	0.32	0.81		CCG/CGG	1.71	0.30	1.00		GAC/GTC	0.99	0.40	0.50	1.00	1.20	0.68	0.80
GCA/TGC	0.16	0.95	0.56		GGA/TCC	0.85	0.89	0.87		AGA/TCT	1.35	0.20	1.01	0.33	0.60	0.79	0.71
TAA/TTA	0.00	0.95	0.47		ACC/GGT	0.43	1.19	0.81		GAA/TTC	1.40	0.40	1.64	0.17	0.00	0.68	0.71
ACT/AGT	0.66	0.16	0.41		GCA/TGC	0.43	0.89	0.66		ACC/GGT	1.29	0.40	0.88	0.33	0.60	0.23	0.62
AAT/ATT	0.16	0.63	0.40		ATG/CAT	0.85	0.30	0.58		ACG/CGT	0.79	1.39	0.13	0.50	0.60	0.11	0.59
CAG/CTG	0.33	0.32	0.32		AGC/GCT	0.43	0.30	0.36		ACA/TGT	0.14	0.20	1.89	0.08	0.60	0.45	0.56
AGC/GCT	0.33	0.32	0.32		GTA/TAC	0.00	0.60	0.30		ATC/GAT	0.32	0.59	0.38	0.25	0.00	0.57	0.35
GAC/GTC	0.33	0.32	0.32		GAC/GTC	0.43	0.00	0.21		TCA/TGA	0.32	0.20	0.38	0.00	0.30	0.57	0.29
CCG/CGG	0.16	0.47	0.32		ACT/AGT	0.43	0.00	0.21		AAC/GTT	0.14	0.00	0.88	0.17	0.30	0.00	0.25
GCC/GGC	0.00	0.47	0.24		CGC/GCG	0.00	0.30	0.15		ATG/CAT	0.25	0.20	0.25	0.08	0.00	0.57	0.22
ATA/TAT	0.00	0.47	0.24		GCC/GGC	0.00	0.30	0.15		ATA/TAT	0.14	0.40	0.50	0.17	0.00	0.00	0.20
GTA/TAC	0.33	0.00	0.16		AAC/GTT	0.00	0.30	0.15		AAT/ATT	0.25	0.00	0.13	0.17	0.30	0.11	0.16
CTA/TAG	0.33	0.00	0.16		AGG/CCT	0.00	0.00	0.00		ACT/AGT	0.11	0.59	0.13	0.00	0.00	0.00	0.14
CGA/TCG	0.16	0.16	0.16		CGA/TCG	0.00	0.00	0.00		TAA/TTA	0.18	0.00	0.13	0.41	0.00	0.00	0.12
CGC/GCG	0.00	0.00	0.00		ACG/CGT	0.00	0.00	0.00		GTA/TAC	0.07	0.20	0.38	0.00	0.00	0.00	0.11
ACG/CGT	0.00	0.00	0.00		CTA/TAG	0.00	0.00	0.00		CTA/TAG	0.09	0.20	0.13	0.00	0.00	0.00	0.07

Tetramers																	
AAGA/TCTT	0.33	0.47	0.40		TAAA/TTTA	0.85	0.89	0.87		CCTC/GAGG	0.09	0.40	0.50	0.17	0.00	0.79	0.32
AAAC/GTTT	0.33	0.32	0.32		TTAA/TTAA	0.85	0.30	0.58		AGGA/TCCT	0.14	0.00	0.13	0.17	0.60	0.57	0.27
GAAA/TTTC	0.33	0.32	0.32		AAGA/TCTT	0.43	0.60	0.51		CATC/GATG	0.27	0.00	0.50	0.25	0.00	0.57	0.26
AGGA/TCCT	0.16	0.16	0.16		AAAG/CTTT	0.00	0.60	0.30		CACG/CGTG	0.09	0.20	0.13	0.08	0.60	0.45	0.26
CAAA/TTTG	0.16	0.16	0.16		AGAT/ATCT	0.00	0.60	0.30		AAAG/CTTT	0.14	0.20	0.00	0.08	0.90	0.23	0.26
CATA/TATG	0.16	0.16	0.16		AAAT/ATTT	0.43	0.00	0.21		ATGC/GCAT	0.00	0.00	0.38	0.33	0.00	0.79	0.25
AAAG/CTTT	0.00	0.32	0.16		AATT/AATT	0.43	0.00	0.21		CATA/TATG	0.14	0.00	0.50	0.41	0.30	0.11	0.24
AACA/TGTT	0.00	0.32	0.16		ATTA/TAAT	0.43	0.00	0.21		TCCA/TGGA	0.11	0.00	0.50	0.50	0.00	0.34	0.24
ACAA/TTGT	0.00	0.32	0.16		CCTC/GAGG	0.43	0.00	0.21		CTGC/GCAG	0.02	0.59	0.38	0.33	0.00	0.11	0.24
GAGC/GCTC	0.16	0.00	0.08		TCTG/CAGA	0.43	0.00	0.21		CTCC/GGAG	0.07	0.20	0.13	0.17	0.60	0.23	0.23

Ara (*Arabidopsis thaliana*), Bra (*Brassica napus*), Lyc (*Solanum lycopersicum),* Sol (*Solanum tuberosum*), Ory (*Oryza sativa),* Sor (*Sorghum bicolor*)*,* Tri (*Triticum aestivum*), Zea (*Zea mays*)*,* Sac (*Saccharum officinarum*) and Hor (*Hordeum vulgare*).

**Table 5:** Distribution of penta- to decamers motifs, percentage occurrence per species and average occurrence per family.

	Brassicaceae					Solanaceae					Poaceae						
	Ara	Bra	Average			Lyc	Sol	Average			Ory	Sor	Tri	Zea	Sac	Hor	Average
Pentamers																	
GAAAA/TTTTC	0.16	0.47	0.32		AAAAT/ATTTT	0.85	0.30	0.58		CTCTC/GAGAG	0.34	0.59	0.00	0.25	0.30	0.68	0.36
AAAAT/ATTTT	0.16	0.32	0.24		AAAAG/CTTTT	0.85	0.00	0.43		GAGGA/TCCTC	0.32	0.00	0.38	0.17	0.00	0.57	0.24
AAAAC/GTTTT	0.00	0.47	0.24		AGAAG/CTTCT	0.43	0.30	0.36		CTTCC/GGAAG	0.07	0.20	0.25	0.17	0.60	0.11	0.23
CAAAA/TTTTG	0.33	0.00	0.16		ATAAA/TTTAT	0.43	0.30	0.36		GGAGA/TCTCC	0.25	0.20	0.13	0.33	0.00	0.34	0.21
GAATC/GATTC	0.00	0.32	0.16		GAAAA/TTTTC	0.43	0.30	0.36		AGGAG/CTCCT	0.29	0.20	0.13	0.33	0.00	0.23	0.20
AAATA/TATTT	0.16	0.00	0.08		CAAAC/GTTTG	0.00	0.60	0.30		AGAGG/CCTCT	0.32	0.00	0.25	0.17	0.00	0.34	0.18
ACAAA/TTTGT	0.16	0.00	0.08		AAATA/TATTT	0.43	0.00	0.21		CTCCC/GGGAG	0.16	0.00	0.13	0.17	0.60	0.00	0.18
ACAAC/GTTGT	0.16	0.00	0.08		AAATC/GATTT	0.43	0.00	0.21		CACCA/TGGTG	0.00	0.00	0.38	0.33	0.30	0.00	0.17
ACTAG/CTAGT	0.16	0.00	0.08		AACTG/CAGTT	0.43	0.00	0.21		AGAAG/CTTCT	0.09	0.20	0.25	0.00	0.00	0.45	0.17
TGTTC/GAACA	0.16	0.00	0.08		AATAA/TTATT	0.43	0.00	0.21		AGGGG/CCCCT	0.18	0.00	0.25	0.08	0.00	0.45	0.16
Hexamers																	
GATGAA/TTCATC	0.33	0.16	0.24		GGTGGA/TCCACC	0.00	2.38	1.19		CGGCGA/TCGCCG	0.38	0.20	0.13	0.25	0.30	0.11	0.23
AAAACA/TGTTTT	0.00	0.47	0.24		GAAGTA/TACTTC	0.85	0.60	0.72		GCACCA/TGGTGC	0.09	0.00	0.25	0.17	0.60	0.00	0.19
AAGGAG/CTCCTT	0.33	0.00	0.16		AGCAGG/CCTGCT	0.85	0.30	0.58		AGGCGG/CCGCCT	0.25	0.20	0.13	0.25	0.00	0.23	0.17
AGCCTC/GAGGCT	0.33	0.00	0.16		CAGCAA/TTGCTG	0.43	0.60	0.51		CCGACG/CGTCGG	0.09	0.00	0.00	0.17	0.60	0.11	0.16
ATCACC/GGTGAT	0.33	0.00	0.16		CCAACA/TGTTGG	0.85	0.00	0.43		CCGTCG/CGACGG	0.18	0.00	0.13	0.17	0.30	0.11	0.15
ATGAAG/CTTCAT	0.33	0.00	0.16		CCTATC/GATAGG	0.85	0.00	0.43		GCCTCC/GGAGGC	0.18	0.40	0.13	0.17	0.00	0.00	0.14
CATCAC/GTGATG	0.33	0.00	0.16		GGATGA/TCATCC	0.85	0.00	0.43		GCCACC/GGTGGC	0.02	0.40	0.00	0.00	0.30	0.11	0.14
CCTCCA/TGGAGG	0.33	0.00	0.16		AGGAAG/CTTCCT	0.43	0.30	0.36		CGGCGC/GCGCCG	0.05	0.59	0.00	0.17	0.00	0.00	0.13
CCTGAG/CTCAGG	0.33	0.00	0.16		ATGAAG/CTTCAT	0.43	0.30	0.36		CGACGC/GCGTCG	0.07	0.40	0.00	0.33	0.00	0.00	0.13
GAATCC/GGATTC	0.33	0.00	0.16		CAACCT/AGGTTG	0.43	0.30	0.36		GGAGCC/GGCTCC	0.00	0.20	0.13	0.17	0.30	0.00	0.13
Heptamers																	
ACACAAA/TTTGTGT	0.33	0.00	0.16		CTTCTCT/AGAGAAG	0.85	0.00	0.43		CCGCCGC/GCGGCGG	0.18	0.20	0.00	0.00	0.00	0.11	0.08
GAGAGAA/TTCTCTC	0.16	0.16	0.16		GATCTCC/GGAGATC	0.85	0.00	0.43		CGCCGCC/GGCGGCG	0.02	0.20	0.25	0.00	0.00	0.00	0.08
AGAGAGA/TCTCTCT	0.00	0.32	0.16		AAAAAAT/ATTTTTT	0.43	0.30	0.36		CCGGCGA/TCGCCGG	0.00	0.40	0.00	0.00	0.00	0.00	0.07
AATTACA/TGTAATT	0.16	0.00	0.08		AAATTTA/TAAATTT	0.43	0.30	0.36		CCGCCGA/TCGGCGG	0.00	0.00	0.00	0.08	0.30	0.00	0.06
ATGAGTG/CACTCAT	0.16	0.00	0.08		TCAACTA/TAGTTGA	0.00	0.60	0.30		CGGCAGG/CCTGCCG	0.02	0.00	0.00	0.00	0.30	0.00	0.05
CAGCGAC/GTCGCTG	0.16	0.00	0.08		TTTTTTG/CAAAAAA	0.00	0.60	0.30		AAAATGA/TCATTTT	0.00	0.00	0.00	0.00	0.30	0.00	0.05
CATTCAA/TTGAATG	0.16	0.00	0.08		AATTGAG/CTCAATT	0.43	0.00	0.21		ACGCAAG/CTTGCGT	0.00	0.00	0.00	0.00	0.30	0.00	0.05
CCTCTCT/AGAGAGG	0.16	0.00	0.08		AGAAACA/TGTTTCT	0.43	0.00	0.21		AGCAGAG/CTCTGCT	0.00	0.00	0.00	0.00	0.30	0.00	0.05
CTCAACT/AGTTGAG	0.16	0.00	0.08		ATCGCCG/CGGCGAT	0.43	0.00	0.21		CACGCCG/CGGCGTG	0.00	0.00	0.00	0.00	0.30	0.00	0.05
TCTCAAA/TTTGAGA	0.16	0.00	0.08		ATGATTC/GAATCAT	0.43	0.00	0.21		CACTGCG/CGCAGTG	0.00	0.00	0.00	0.00	0.30	0.00	0.05
Octamers																	
ATGTATGA/TCATACAT	0.16	0.00	0.08		AAGAAAAA/TTTTTCTT	0.00	0.30	0.15		GAAGTCAA/TTGACTTC	0.00	0.00	0.13	0.00	0.00	0.00	0.02
CCCCTTCT/AGAAGGGG	0.16	0.00	0.08		TTTCTCTC/GAGAGAAA	0.00	0.30	0.15		GCGACCGA/TCGGTCGC	0.00	0.00	0.13	0.00	0.00	0.00	0.02
CTTGTTCC/GGAACAAG	0.16	0.00	0.08		AAAAAAAC/GTTTTTTT	0.00	0.00	0.00		CCGCACGC/GCGTGCGG	0.00	0.00	0.00	0.00	0.00	0.11	0.02
GAAGCAAG/CTTGCTTC	0.16	0.00	0.08		ACGGGCGA/TCGCCCGT	0.00	0.00	0.00		CCTATCTA/TAGATAGG	0.00	0.00	0.00	0.00	0.00	0.11	0.02
AAAAAAAC/GTTTTTTT	0.00	0.16	0.08		AGAAAAAA/TTTTTTCT	0.00	0.00	0.00		CAAGAAGC/GCTTCTTG	0.05	0.00	0.00	0.00	0.00	0.00	0.01
AGAAAAAA/TTTTTTCT	0.00	0.16	0.08		ATCAGGGA/TCCCTGAT	0.00	0.00	0.00		ACGGGCGA/TCGCCCGT	0.02	0.00	0.00	0.00	0.00	0.00	0.00
TCTTTGTG/CACAAAGA	0.00	0.16	0.08		ATGATGTA/TACATCAT	0.00	0.00	0.00		ATCAGGGA/TCCCTGAT	0.02	0.00	0.00	0.00	0.00	0.00	0.00
AAGAAAAA/TTTTTCTT	0.00	0.00	0.00		ATGTATGA/TCATACAT	0.00	0.00	0.00		ATGATGTA/TACATCAT	0.02	0.00	0.00	0.00	0.00	0.00	0.00
ACGGGCGA/TCGCCCGT	0.00	0.00	0.00		CAAGAAGC/GCTTCTTG	0.00	0.00	0.00		TCAAATTT/AAATTTGA	0.02	0.00	0.00	0.00	0.00	0.00	0.00
ATCAGGGA/TCCCTGAT	0.00	0.00	0.00		CCCCTTCT/AGAAGGGG	0.00	0.00	0.00		TGGGCTTG/CAAGCCCA	0.02	0.00	0.00	0.00	0.00	0.00	0.00
Nonamers																	
AAGATGAAG/CTTCATCTT	0.16	0.00	0.08		ACTCCTTCA/TGAAGGAGT	0.00	0.30	0.15		ACGACTACG/CGTAGTCGT	0.00	0.00	0.00	0.00	0.30	0.00	0.05
AATGGGTGG/CCACCCATT	0.16	0.00	0.08		CAAATTACC/GGTAATTTG	0.00	0.30	0.15		AGCGAAGAA/TTCTTCGCT	0.00	0.00	0.00	0.00	0.30	0.00	0.05
AGAAGGAAG/CTTCCTTCT	0.16	0.00	0.08		CAGACTATT/AATAGTCTG	0.00	0.30	0.15		AGCACCAGC/GCTGGTGCT	0.00	0.20	0.00	0.00	0.00	0.00	0.03
ATGGGTGAC/GTCACCCAT	0.16	0.00	0.08		CTTCTTATC/GATAAGAAG	0.00	0.30	0.15		GGTGGTATG/CATACCACC	0.00	0.20	0.00	0.00	0.00	0.00	0.03
GAAGGAGAA/TTCTCCTTC	0.16	0.00	0.08		AAAAAAAAC/GTTTTTTTT	0.00	0.00	0.00		ACCCTCTCC/GGAGAGGGT	0.00	0.00	0.13	0.00	0.00	0.00	0.02
GAGAAGAAG/CTTCTTCTC	0.16	0.00	0.08		AACAGGAGA/TCTCCTGTT	0.00	0.00	0.00		CCGCTGGAT/ATCCAGCGG	0.00	0.00	0.13	0.00	0.00	0.00	0.02
GAGGAAGAA/TTCTTCCTC	0.16	0.00	0.08		AAGATGAAG/CTTCATCTT	0.00	0.00	0.00		GCTGTGACC/GGTCACAGC	0.00	0.00	0.13	0.00	0.00	0.00	0.02
GAGGAAGAG/CTCTTCCTC	0.16	0.00	0.08		AATGGGTGG/CCACCCATT	0.00	0.00	0.00		ACCACCAGC/GCTGGTGGT	0.00	0.00	0.00	0.00	0.00	0.11	0.02
TATAATTCG/CGAATTATA	0.16	0.00	0.08		ACAGCAACA/TGTTGCTGT	0.00	0.00	0.00		ACCACGGAC/GTCCGTGGT	0.00	0.00	0.00	0.00	0.00	0.11	0.02
TCTTCGTCT/AGACGAAGA	0.16	0.00	0.08		ACCACCAGC/GCTGGTGGT	0.00	0.00	0.00		CCATCCTTA/TAAGGATGG	0.00	0.00	0.00	0.00	0.00	0.11	0.02
Decamers																	
ACTTTGAGTG/CACTCAAAGT	0.16	0.00	0.08		AAAAAGAAAA/TTTTCTTTTT	0.00	0.00	0.00		AAAAAGAAAA/TTTTCTTTTT	0.00	0.00	0.00	0.00	0.30	0.00	0.05
CAAAGTCACT/AGTGACTTTG	0.16	0.00	0.08		ACTTTGAGTG/CACTCAAAGT	0.00	0.00	0.00		CCACGCGTCG/CGACGCGTGG	0.23	0.00	0.00	0.00	0.00	0.00	0.04
TTTTTTTTCT/AGAAAAAAAA	0.00	0.16	0.08		AGCCCCAACG/CGTTGGGGCT	0.00	0.00	0.00		TTTTTTTTCT/AGAAAAAAAA	0.00	0.00	0.13	0.00	0.00	0.00	0.02
AAAAAGAAAA/TTTTCTTTTT	0.00	0.00	0.00		ATCTCCGCCG/CGGCGGAGAT	0.00	0.00	0.00		AGCCCCAACG/CGTTGGGGCT	0.05	0.00	0.00	0.00	0.00	0.00	0.01

Ara (*Arabidopsis thaliana*), Bra (*Brassica napus*), Lyc (*Solanum lycopersicum),* Sol (*Solanum tuberosum*), Ory (*Oryza sativa),* Sor(*Sorghum bicolor*)*,* Tri (*Triticum aestivum*), Zea (*Zea mays*)*,* Sac (*Saccharum officinarum*) and Hor (*Hordeum vulgare*).
